# The critical BMI hypothesis for puberty initiation and the gender prevalence difference: Evidence from an epidemiological survey in Beijing, China

**DOI:** 10.3389/fendo.2022.1009133

**Published:** 2022-10-25

**Authors:** Meijuan Liu, Bingyan Cao, Qipeng Luo, Qiao Wang, Min Liu, Xuejun Liang, Di Wu, Wenjing Li, Chang Su, Jiajia Chen, Chunxiu Gong

**Affiliations:** ^1^ Department of Endocrinology, Genetics and Metabolism, National Center for Children’s Health, Beijing Children’s Hospital, Capital Medical University, Beijing, China; ^2^ Department of Pain Medicine, Peking University Third Hospital, Beijing, China

**Keywords:** children, body mass index, central obesity, precocious puberty, gender

## Abstract

**Background:**

Although previous studies suggested that there is a certain level of body fat mass before puberty can be initiated, most studies have focused on girls.

**Objective:**

To investigate the relationship between precocious puberty and physical growth in school-aged children in Beijing, China.

**Methods:**

7590 Chinese children (3591 girls and 3999 boys) aged 6–11 years were recruited in Beijing, China. Body mass index (BMI) categories were defined by WHO Child Growth Standards and central obesity were defined by sex-specific waist-to-height ratio cut-offs (≥0.46 for girls, ≥0.48 for boys). Sexual development was assessed using Tanner criteria.

**Results:**

The prevalence of general obesity and central obesity among boys was higher than that in girls. Girls had a significantly higher precocious puberty rate than boys (5.93% vs. 0.87%), particularly in those aged 7 years old (9.20%). Children in the general obesity and central obesity groups have a higher prevalence of precocious puberty and earlier median ages for the attainment of Tanner B_2_/T_2_. For girls with Tanner stages≥II at 6-year-old and 7-year-old, the mean BMI was equivalent to the 50th centile of a normal 9.9-year-old and 11.9-year-old girl, respectively. The mean BMI of boys with Tanner stages≥II at 7-year-old and 8-year-old was correspondent to the 50th centile of a normal 14-year-old and 15.3-year-old boy, respectively. For girls, general obesity appears to contribute to the risk of the development of precocious puberty to a greater extent than central obesity does. For boys, central obesity, but not general obesity, was an independent risk factor for precocious puberty.

**Conclusions:**

The prevalence of childhood obesity and precocious puberty was high in China. Precocious puberty was correlated with a large BMI. Boys had a higher threshold of BMI for puberty development than girls. Children with precocious puberty, particularly those with central obesity, should be aware of adverse cardiovascular events.

## Introduction

Puberty is a critical period between childhood and adulthood during which adolescents reach sexual maturity and become capable of reproduction (1). The ages of transition into a higher Tanner stage of breast, pubic hair, and testicle development are maturational milestones commonly used to describe the initiation and progression of puberty among girls and boys. In recent decades, a growing body of literature reports the earlier development of secondary sexual characteristics in children (2–6). According to the data collected from the National Health and Nutrition Examination Survey (NHANES), the mean age for menarche in the United States declined over time from 13.3 years in girls born before 1920 to 12.4 years in girls born in 1980–1984 (4). In Thailand, the median age of onset of breast development showed a steady decline, from 10.7 years in 1980 to 9.9 years in 1994, and recently to 9.4 years in 1999 (2). It is important to note, that earlier ages of puberty have been reported to be associated with a higher risk of adult disease, including obesity, type 2 diabetes mellitus, metabolic syndrome, depression, cardiovascular disease, cancer, etc. (7–9). Thus, obtaining up-to-date pubertal data and understanding modifiable factors influencing pubertal timing could have important public health implications.

Indeed, puberty is a complex physical process, which involves a complicated interplay between genetic, nutritional, environmental, and socio-economic factors (10). Although the mechanisms controlling the onset and tempo of puberty are still largely unknown, accumulating research has confirmed the important role of obesity in sexual development (11–14). In recent years, the decreasing age of pubertal onset has paralleled the rising prevalence of pediatric obesity (15), and multiple studies have demonstrated that childhood overweight/obesity is associated with earlier timing or faster progression of pubertal development (16–18). For instance, overweight and obese girls were more likely to attain pubertal milestones earlier compared with normal-weight children [overweight girls: -5.5 months, 95% confidence interval (CI): -7.1, -3.9 months; obese girls: -5.2 months, 95% CI: -7.1, -3.4 months] (17). Furthermore, previous studies indicated that body weight was involved in puberty onset and maintenance (19, 20). Girls who had earlier onset of puberty had markedly higher age-normalized body mass index (BMI) (21), whereas girls who had low body weight or body fat were often accompanied by delayed menarche and menstrual irregularities (20, 22). That is, a certain threshold of body weight and fat has to be reached before puberty can be initiated and maintained. However, most of these studies focus on girls, and results on boys seem to be inconsistent (16, 17, 23).

As well as obesity, body fat distribution has lately been found to be associated with sexual development. Boys and girls with a high-level body fat percentage (24) and waist-to-height ratio (WHtR) (25) had increased risks of earlier puberty in comparison with those with a low-level body fat percentage and WHtR. However, in the majority of studies, overweight/obesity is defined by BMI level, which was not effective for providing information on fat distribution. There are, indeed, only a few studies have examined the association between abdominal obesity and puberty onset.

With the above-mentioned points in mind, this study aimed to estimate the prevalence and distribution of childhood precocious puberty, especially in different age and weight categories; and to explore the associations between childhood general obesity/central obesity with puberty onset.

## Methods

### Study population

This was a cross-sectional, school-based study, carried out in Beijing, the capital of China. The data presented here were obtained from the survey on Students’ Constitution and Health. Children were excluded if they were taking medication (such as glucocorticoid) that could cause precocious puberty or had unilateral mammary development, or a preexisting diagnosis of organic disease (such as ovarian tumors), or chronic diseases (such as chronic kidney disease), or genital abnormalities (such as cryptorchidism). A total sample of 7590 students (3591 girls and 3999 boys) aged 6–11 years were enrolled in this study. For general obesity, children were classified into five categories: severe thinness (n=151), thinness (n=520), normal (n=3787), overweight (n=1346), and obesity (n=1786). For central obesity, children were classified into two categories: non-central obesity (n=4908) and central obesity (n=2682). All students completed a physical examination. Written informed consent was obtained from a parent or guardian on behalf of each child and the study was approved by the ethics committee of Beijing Children’s Hospital, Capital Medical University.

### Physical examination

All children underwent a standardized series of physical examinations that were conducted by trained pediatric endocrinologists. Body weight (to the nearest 0.1 kg) was measured using a scale (Tanita HA 503, Tanita Corporation, Tokyo, Japan) with subjects wearing only lightweight clothing and without shoes. Height (to the nearest 0.1 cm) was assessed in the erect position without shoes by a portable Seca 213 stadiometer (Seca GmbH, Hamburg, Germany). Waist circumference (WC, to the nearest 0.1 cm) was obtained with an inelastic measuring tape at the midpoint of the horizontal line between the lowest rib margin and the iliac crest. Two measurements (measurement error ≤ 0.1 kg/0.1 cm) were taken and the average was calculated for the analysis. BMI was calculated as children’s weight (kg) divided by the square of height (m^2^). Children were classified into five categories: severe thinness, thinness, normal, overweight, and obesity according to sex-age-specific BMI cut-offs recommended by the WHO Child Growth Standards (http://www.who.int/growthref/en/) as follows: severe thinness if BMI<−3 standard deviation (SD), thinness if BMI<−2 SD, overweight if BMI>+1SD, and obesity if BMI>+2SD (26, 27). WHtR was calculated as WC (cm) divided by height (cm), figures equal to or greater than 0.46 and 0.48 were considered central obesity for girls and boys, respectively (28).

The puberty assessment of children was evaluated by professional pediatric endocrinology physicians. In girls, breast development was evaluated by inspection combined with palpation. In boys, testicular volume was estimated by palpation to the nearest 1 mL using Prader’s orchidometer. The pubertal stages of breast, pubic hair, and testicular volume were graded from I (prepubertal) to V (fully mature) using the Tanner staging method (29, 30). Precocious puberty was defined as the occurrence of Tanner stage II or above for breast (B_2_) or pubic hair development (PH_2_) before 8 years in girls, and Tanner stage II or above for testicle development (T_2_, volume ≥4 mL) or PH_2_ before 9 years in boys (31). Sexual development ≥B_2_/PH_2_ in girls and T_2_/PH_2_ in boys were defined as Tanner stage II or above for breast/pubic hair development in girls and testicle/pubic hair development in boys (25).

### Statistical analysis

Categorical variables were presented as frequency (percentage), and continuous variables were presented as mean  ± SD. Comparisons of continuous variables were performed using Student t-tests, and comparisons of categorical variables were performed using Chi-squared tests with Fisher’s exact statistic. The detection rates of Tanner stages≥II in different ages and obesity categories were directly calculated. Probit analysis was used for estimating the median age of the population at entry into Tanner stage II or greater for breast, pubic hair, and testicular development. Odds ratios (ORs) with 95% CIs were calculated to assess the associations between precocious puberty, BMI categories, and central obesity. All the raw data were analyzed by IBM SPSS Statistic package version 21 (IBM Corp., Armonk, New York, USA), *p* value<0.05 was considered statistically significant.

## Results

### Subject characteristics

In total, 7590 students (girls: 3591, boys: 3999) aged 6–11 years were assessed in this study, and the basic characteristics were summarized in **Table 1**. The age distribution was similar for boys and girls (*p*>0.05). Boys had higher weight, height, WC, BMI, and WHtR than girls (*p* all <0.05). The proportion of general obesity and central obesity were markedly higher in boys than in girls (general obesity: 32.28% vs. 13.78%, central obesity: 39.93% vs. 30.21%).

**Table 1:** Basic characteristics of the subjects.

Variables	Total N=7590	Girls N=3591	Boys N=3999	*p*-value
**Age (years)**				0.672
** 6**	1238 (16.31%)	602 (16.76%)	636 (15.90%)	
** 7**	1491 (19.64%)	696 (19.38%)	795 (19.88%)	
** 8**	1462 (19.26%)	705 (19.63%)	757 (18.93%)	
** 9**	1448 (19.08%)	674 (18.77%)	774 (19.35%)	
** 10**	1062 (13.99%)	509 (14.17%)	553 (13.83%)	
** 11**	889 (11.71%)	405 (11.28%)	484 (12.10%)	
**Weight (kg)**	34.49 ± 12.87	32.62 ± 11.67	36.18 ± 13.65	<0.05
**Height (cm)**	135.91 ± 11.65	135.27 ± 11.86	136.48 ± 11.43	<0.05
**WC (cm)**	61.68 ± 11.48	58.82 ± 10.02	64.26 ± 12.08	<0.05
**BMI (kg/m^2^)**	18.18 ± 4.51	17.37 ± 3.92	18.90 ± 4.87	<0.05
**WHtR**	0.86 ± 0.23	0.83 ± 0.30	0.88 ± 0.13	<0.05
**BMI category**				<0.05
** Severe thinness**	151 (1.99%)	27 (0.75%)	124 (3.10%)	
** Thinness**	520 (6.85%)	124 (3.45%)	396 (9.90%)	
** Normal**	3787 (49.89%)	2302 (64.10%)	1485 (37.13%)	
** Overweight**	1346 (17.73%)	643 (17.91%)	703 (17.58%)	
** Obesity**	1786 (23.53%)	495 (13.78%)	1291 (32.28%)	
**Central obesity category**				<0.05
** Non-central obesity**	4908 (64.66%)	2506 (69.79%)	2402 (60.07%)	
** Central obesity**	2682 (35.34%)	1085 (30.21%)	1597 (39.93%)	

WC, waist circumference; BMI, body mass index; WHtR, waist-to-height ratio. The data were presented as the mean ± SD for continuous variables or as the number (%) for categorical variables. The p-values were obtained from the independent t-test for continuous variables and chi-square tests for categorical variables.

### Prevalence and distribution of sexual development in different age and weight categories

**Figure 1:**
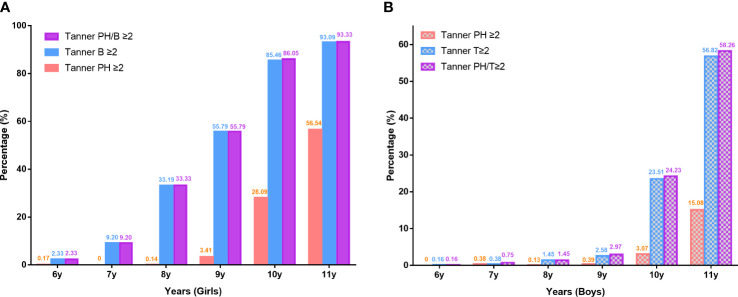
Prevalence of testicle, breast, and pubic hair development according to pubertal stages at different ages in girls **(A)** and boys **(B)**. Ph, pubic hair; T, testicle; B, breast.

The prevalence of sexual development ≥B_2_/PH_2_ in girls and T_2_/PH_2_ in boys at different ages was depicted in **Figure 1**. Prevalence of Tanner stages ≥II was significantly higher in girls than in boys. The prevalence of Tanner stages ≥PH_2_ among girls aged 9–11 years was relatively higher than those in boys (9 years: 3.41% vs. 0.39%, 10 years: 28.09% vs. 3.07%, 11 years: 56.54% vs. 15.08%). At ages 6–11 years, girls had reached a higher proportion of Tanner stages ≥B_2_ than boys with Tanner stages ≥T_2_ (6 years: 2.33% vs. 0.16%, 7 years: 9.20% vs. 0.38%, 8 years: 33.19% vs. 1.45%, 9 years: 55.79% vs. 2.58%, 10 years: 85.46% vs. 23.51%, 11 years: 93.09% vs. 56.82%). Girls had a significantly higher precocious puberty rate than boys. The overall rate of precocious puberty in girls aged <8 years and in boys aged <9 years was 5.93% and 0.87%, respectively. The highest precocious puberty rate for girls and boys was in the 7-year-old group (9.20%) and the 8-year-old group (1.45%), respectively.

**Figure 2:**
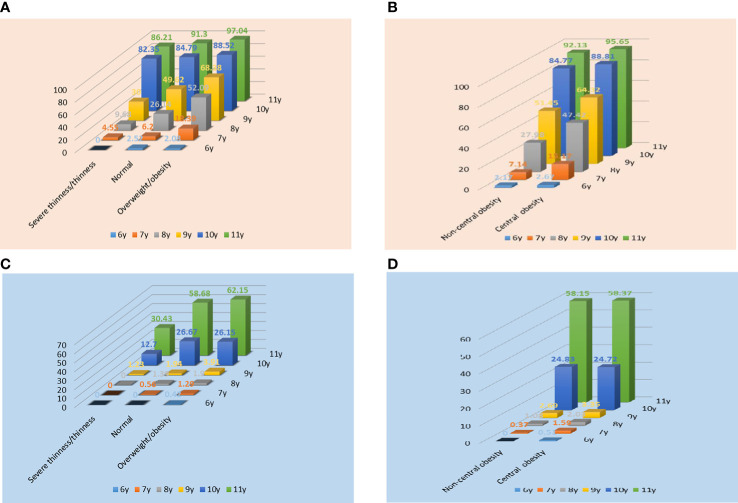
Prevalence of Tanner stages ≥II in different obesity categories in girls **(A, B)** and boys **(C, D)**. In Figure A, C, normal refers to children with normal body mass index.

The prevalence of Tanner stages ≥II in different obesity categories in both genders was also examined. As shown in **Figure 2**, general obesity and central obesity had an obvious influence on sexual development in both genders at different ages. Compared with children with severe thinness/thinness, we found an increased prevalence of Tanner stages ≥II in the normal-weight group in both genders, and an especially high prevalence of Tanner stages ≥II in the overweight/obesity group. Additionally, the prevalence of Tanner stages ≥II in the central obesity group was higher than that in the normal-weight group in both genders at different ages. In both genders, the prevalence of precocious puberty in the overweight/obesity and central obesity group was higher than that in the control group (girls: 11.01% vs. 4.49%, 6.01% vs. 4.93%; boys: 1.31% vs. 0.64%, 1.48% vs. 0.49%).

### The median age of attaining Tanner B_2_/T_2_/PH_2_

To further explore the effects of obesity on puberty onset, we then estimated the median age of both girls and boys for the attainment of Tanner B_2_/T_2_/PH_2_ (**Table 2**). In girls, the median age of B_2_/PH_2_ was 10.13 and 11.14 in the severe thinness/thinness group, 9.20 and 10.73 in the normal-weight group, 8.92 and 10.72 in the overweight/obesity group. A similar decreasing trend with increasing BMI was observed in the median ages of T_2_ (10.83, 10.67, 10.63 years) in boys, although the trend was not significant. This finding indicated that severe thinness/thinness had a greater impact on puberty onset than overweight/obesity. Notably, negative associations between general obesity and median ages of B_2_ and T_2_ were also found in central obesity. For girls, the median age of B_2_ was 9.27 (95% CI=9.17 to 9.38) and 8.84 (95% CI=8.59 to 9.09) years for children in the control group and the central obesity group. For boys, the median age of T_2_ in the control group (10.67, 95% CI=10.56 to 10.79) was older than that in the central obesity group (10.63, 95% CI=10.49 to 10.78). Although the trend for earlier median ages of Tanner B_2_/T_2_/PH_2_ was similar in girls and boys, it was more profound in girls.

**Table 2:** Median age (95% CI) of attainment of different pubertal stages according to probit analysis.

Variables	Girls			Boys		
BMI category	B_2_	PH_2_	B_2_/PH_2_	T_2_	PH_2_	T_2_/PH_2_
**Severe thinness/thinness**	10.13 (9.70-10.55)	11.14 (10.56-11.72)	10.31 (9.53-10.50)	10.83 (10.56-11.11)	/	10.83 (10.56-11.11)
**Normal**	9.20 (9.09-9.31)	10.73 (10.63-10.84)	9.49 (9.39-9.60)	10.67 (10.50-10.83)	10.83 (10.37-11.28)	10.68 (10.52-10.84)
**Overweight/obesity**	8.92 (8.70-9.15)	10.72 (10.60-10.85)	9.81 (9.63-9.99)	10.63 (10.51-10.75)	11.10 (10.95-11.25)	10.71 (10.60-10.80)
**Central obesity category**						
**Non-central obesity**	9.27 (9.17-9.38)	10.74 (10.64-10.84)	9.61 (9.52-9.71)	10.67 (10.56-10.79)	10.92 (10.61-11.23)	10.69 (10.58-10.81)
**Central obesity**	8.84 (8.59-9.09)	10.74 (10.60-10.89)	9.65 (9.44-9.86)	10.63 (10.49-10.78)	11.11 (10.93-11.28)	10.71 (10.59-10.84)
**Total**	9.20 (9.10-9.30)	10.74 (10.66-10.83)	9.62 (9.53-9.71)	10.65 (10.56-10.74)	10.81 (10.73-10.88)	10.70 (10.62-10.77)

B_2_, Tanner stage II for breast development; PH_2_, Tanner stage II for pubic hair development; T_2_, Tanner stage II for testicle development with testicular volume ≥4mL; BMI, body mass index.

### Relationship between precocious puberty and physical growth

**Figure 3:**
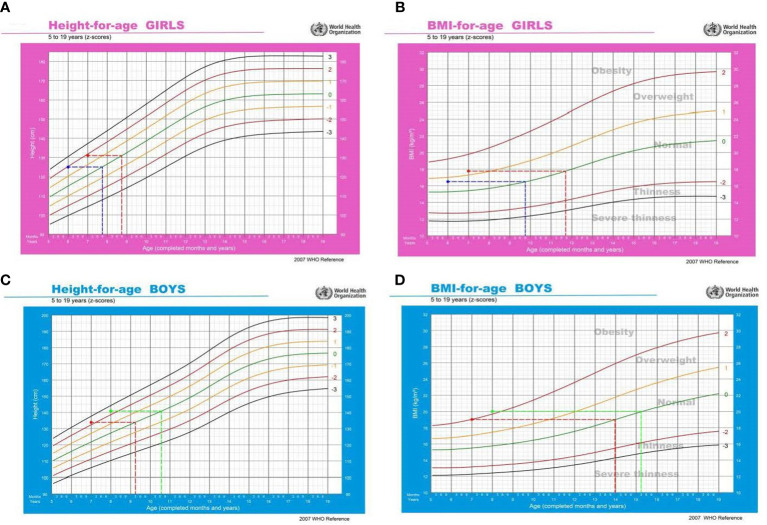
Relationship between precocious puberty and physical growth. In Figures **(A, B)**, the blue line refers to girls with Tanner stages≥II at 6-year-old, and the red line refers to girls with Tanner stages≥II at 7-year-old. In Figures **(C, D)**, the red line refers to boys with Tanner stages≥II at 7-year-old, and the green line refers to boys with Tanner stages≥II at 8-year-old.

In both genders, precocious puberty was correlated with a large physical growth, especially for BMI. As illustrated in **Figure 3A**, the mean height of girls with Tanner stages≥II at 6-year-old and 7-year-old was 125.01 ± 9.71 cm and 131.46 ± 5.69 cm, which was equivalent to the 50th centile of a normal 7.9-year-old and 8.9-year-old girl, respectively (**Figure 3A**). For boys with Tanner stages ≥II at 7-year-old and 8-year-old, the mean height was 133.93 ± 9.71 cm and 141.15 ± 7.07 cm, which was correspondent to the 50th centile of a normal 9.3-year-old and 10.6-year-old boy, respectively (**Figure 3C**). Similar phenomena were observed for BMI. For girls with Tanner stages ≥II at 6-year-old and 7-year-old, the mean BMI was 16.56 ± 4.11 kg/m^2^ and 17.71 ± 2.93 kg/m^2^, which was equivalent to the 50th centile of a normal 9.9-year-old and 11.9-year-old girl, respectively (**Figure 3B**). The mean BMI of boys with Tanner stages ≥II at 7-year-old and 8-year-old was 19.04 ± 3.13 kg/m^2^ and 20.05 ± 4.29 kg/m^2^, which was correspondent to the 50th centile of a normal 14-year-old and 15.3-year-old boy, respectively (**Figure 3D**). Taken together, it appears that physical growth, especially BMI must be kept above a certain threshold level to initiate puberty development. The threshold was much higher in boys than in girls, which was in accordance with the above observations (higher prevalence of obesity and lower rate of precocious puberty in boys).

### Relationship between different types of obesity and the risk of precocious puberty

We further explored the relationship between different types of obesity and the risk of precocious puberty. As shown in **Table 3**, for girls, both general obesity and central obesity were independently associated with an increased risk of precocious puberty. General obesity appears to contribute to the risk of development of precocious puberty to a greater extent than central obesity does (general obesity: OR=2.46, 95% CI 1.33–4.55; central obesity: OR=1.81, 95% CI 1.14–2.89; respectively). For boys, central obesity, but not general obesity, was an independent risk factor for precocious puberty(OR=2.93, 95% CI 1.04–8.26, *p*<0.05).

**Table 3:** Associations between different types of obesity and the risk of precocious puberty.

Puberty	General obesity			Central obesity		
	Normal, N (%)	Obesity, N (%)	OR (95% CI)	Normal, N (%)	Obesity, N (%)	OR (95% CI)
**Total**						
** Normal**	1830 (97.44%)	720 (96.90%)	Reference	2324 (97.77%)	1069 (96.31%)	Reference
** Precocious**	48 (2.56%)	23 (3.10%)	1.22 (0.74-2.02)	53 (2.23%)	41 (3.69%)	**1.68 (1.11-2.54)**
**Girls**						
** Normal**	894 (95.41%)	127 (89.44%)	Reference	887 (94.97%)	333 (91.23%)	Reference
** Precocious**	43 (4.59%)	15 (10.56%)	**2.46 (1.33-4.55)**	47 (5.03%)	32 (8.77%)	**1.81 (1.14-2.89)**
**Boys**						
** Normal**	936 (99.47%)	593 (98.67%)	Reference	1437 (99.58%)	736 (98.79%)	Reference
** Precocious**	5 (0.53%)	8 (1.33%)	2.53 (0.82-7.76)	6 (0.42%)	9 (1.21%)	**2.93 (1.04-8.26)**

Statistically significant (p < 0.05) were marked in bold.

## Discussion

In this cross-sectional study with a large sample of Chinese children aged 6–11 years in Beijing, we found that the prevalence of general obesity and central obesity were 13.78% and 30.21% in girls, 32.28% and 39.93% in boys, respectively. It is clearly seen that with the rapid economic development and urbanization, the prevalence of childhood obesity in China has reached epidemic proportions. In our present study, the overall rate of precocious puberty in girls aged <8 years and in boys aged <9 years was 5.93% and 0.87%, respectively. The highest precocious puberty rate for girls and boys was in the 7-year-old group (9.20%) and 8-year-old group (1.45%), respectively. The prevalence of precocious puberty observed in this study was significantly higher than that found in previous studies. In a cross-sectional epidemiological study conducted in 6 representative geographical areas in China in 2013, breast development at 7-year-old was observed in 2.56% of girls, and testicular volume ≥4 mL at 8-year-old was observed in 0.61% of boys (32). Recently, studies conducted in other cities in China (Shanghai, Zhongshan, Chongqing, etc.) also noted the high prevalence of precocious puberty (girls: 4.8%–23.07%, boys:0.8%–9.53%) (25, 31, 33). Additionally, we found that boys showed a higher rate of obesity, but a lower rate of precocious puberty rate than girls. The reasons for the higher precocious puberty prevalence rate observed in girls are not entirely clear. We speculate that the distinct criteria used in girls (breast, not gonads) and boys (testicle, gonads) may partially be responsible for this difference in prevalence. The enlarged testes in boys were in a stricter sense of puberty initiation.

In addition to the markedly increased prevalence, the age of puberty onset has also significantly advanced. Jaruratanasirikul et al. reported that the median age of Tanner B_2_ varied from 9.9 years in 1994 to 9.6 years in 2012, which indicated the trend toward earlier onset of puberty in Thailand school girls (2). A similar phenomenon was also observed in mainland China. In 2009–2010, Zhu et al. conducted a cross-sectional study in 18707 children in 6 representative cities in China, they found that the median ages of onset of Tanner B_2_ and T_2_ were 9.69 (95% CI: 9.63–9.75) years and 11.25 (95% CI: 11.19–11.30) years, respectively (32). According to the data from the Shanghai Children’s Health, Education and Lifestyle Evaluation (SCHEDULE) study in 2014, the median ages of B_2_ and PH_2_ for girls were 8.62 (95% CI: 8.57–8.67) years and 10.62 (95% CI: 10.53–10.71) years, the median ages of T_2_ and PH_2_ for boys were 13.84 (95% CI: 13.40–14.40) years and 10.46 (95% CI: 10.21–10.78) years (25). The median ages of T_2_ and PH_2_ for boys in our present study were earlier than that found in previous studies in Shanghai (25), which may be attributable to the higher prevalence of central obesity in boys in our study. However, these studies only focused on the age of puberty onset without anthropometric measurements (weight, height, BMI, etc.), and comparative analysis could not be calculated for these cases. Taken together, the high prevalence and earlier age of precocious puberty observed in our results together with our publications revealed an urgent need for scaled-up effective interventions among Chinese children.

Increasing evidence suggests that obesity is closely relevant to precocious puberty, the rise in the prevalence of childhood obesity alongside the rise in early sexual development, especially in girls (17, 21, 23, 34, 35). Four findings of our study confirmed the close links between obesity and precocious puberty in girls. Firstly, this study revealed that the prevalence of precocious puberty in the overweight/obesity and central obesity group was 11.01% and 6.01%, which was significantly higher than that in the normal-weight (4.49%) and non-central obesity groups (4.93%). This finding was in line with the study of Liu et al. among 4058 children aged 6–11 years in Guangdong (31), also, the study of Wei et al. among 11000 children aged 4–12 years in Leshan (36), both of which showed girls who were overweight or obese had a higher prevalence of precocious puberty. Secondly, we observed that overweight/obesity, as well as central obesity, were associated with earlier age at attaining Tanner B_2_. Results from other studies support our results. In a recent cohort and sibling-matched analyses performed by Brix et al., childhood overweight and obesity were associated with earlier puberty in a dose-dependent manner in boys and girls (17). Thirdly, our study also demonstrated that girls with precocious puberty were correlated with a large BMI. For girls with precocious puberty at 6-year-old and 7-year-old, the mean BMI was 16.56 ± 4.11 kg/m^2^ and 17.71 ± 2.93 kg/m^2^, which was equivalent to the 50th centile of a normal 9.9-year-old and 11.9-year-old girl, respectively. As early as the 1970s, Frisch proposed the “critical weight hypothesis”, which stated that a certain body fat depot seems to be required for the process of initiating normal reproductive function (20). Previous studies found that the values of weight, height, WC (33), BMI, and WHtR (32) in precocious puberty children were higher than those in peer normal children. Children with high-level of body fat as well as those with a rapid increase in anthropometric profiles were more sensitive to earlier puberty onset (24). Our results, together with data reported by others, indicated the importance to maintain healthy adiposity status in preventing earlier puberty onset in children. Fourthly, in the present study, general obesity and central obesity were found to as individual risk variables affecting precocious puberty. It is noteworthy that in girls, general obesity appears to contribute to the risk of development of precocious puberty to a greater extent than central obesity does (general obesity: OR=2.46, 95% CI 1.33–4.55; central obesity: OR=1.81, 95% CI 1.14–2.89; respectively). Our results were in keeping with studies by Chen et al. in Shanghai, which demonstrated that more than for central obesity, the risk of precocious puberty in girls rose even more with general obesity (general obesity: OR=9.00, 95% CI 5.60–14.46; central obesity: OR=5.40, 95% CI 4.10–7.12; respectively) (25). However, based on the relatively small number of studies available, more findings about the effect of fat distribution on pubertal development in girls should be done in the future.

As stated above, the link between obesity and precocious puberty in girls is well established. However, observations in boys remain inconclusive (31, 37–40). In our present study, we found that the prevalence of Tanner stages ≥II was significantly higher in the general obesity and central obesity groups for boys aged 6–11 years. Furthermore, boys with general obesity and central obesity started puberty, as assessed by testicular enlargement, significantly earlier compared to normal weight and non-central obese boys. However, Our result differs from previous studies by Lee et al. in US boys, who showed a J-shaped association between weight status with the timing of puberty, that is to say, overweight boys seemed to mature earlier, but obese boys matured later than normal-weight boys (37). This disagreement may be attributable to geographic distribution and racial differences. The mean BMI of boys with Tanner stages ≥II at 7-year-old and 8-year-old was 19.04 ± 3.13 kg/m^2^ and 20.05 ± 4.29 kg/m^2^, which was correspondent to the 50th centile of a normal 14-year-old and 15.3-year-old boy, respectively. While boys with precocious puberty were also correlated with a large BMI, just like girls, the threshold of BMI for puberty development in boys was higher than that in girls. We speculate that the distinct criteria used in girls (breast, not gonads) and boys (testicle, gonads) may partially be responsible for this difference in prevalence. The manifestation of puberty initiation was stricter in boys (testicle, gonads) than in girls (breast, not gonads), as the majority of the breast is composed of fat tissue. This was also consistent with the clinical observations that boys had a significantly higher prevalence of obesity but a lower rate of precocious puberty than girls. Another interesting finding worthy of note was that in boys, central obesity was an independent risk factor, whereas general obesity was not. That is, in boys, WHtR had a greater influence than BMI on precocious puberty. Central obesity has been implicated as one of the most important risk factors in the development of various metabolic diseases including hypertension and cardiovascular diseases. Thus, children with precocious puberty, particularly those with central obesity, should be a cause for concern among physicians regarding possible adverse cardiovascular events.

Several limitations should be considered when interpreting our present findings. First, this was a cross-sectional study and the cause-effect relationship between precocious puberty and obesity could not be determined in our study. Longitudinal investigations are needed to determine the causal direction. Second, although the physical examination was conducted by trained pediatric endocrinologists, the inter-observer bias cannot be ruled out. Third, in our present study, the breast development of girls was evaluated by inspection combined with palpation. It was rather difficult to employ ultrasound diagnosis on a large scale in children taking part in the physical examination. Fourth, our results might not be representative of the population as a whole and should be interpreted with caution.

In conclusion, the prevalence of childhood general obesity, central obesity, and precocious puberty was high in China. Children with general obesity and central obesity were more vulnerable to precocious puberty, but this correlation had gender differences. Boys had a higher threshold of BMI for puberty development than girls. The causal relationship between obesity and precocious puberty and the underlying mechanisms need to be studied in the future.

## Data availability statement

The original contributions presented in the study are included in the article/supplementary material. Further inquiries can be directed to the corresponding author.

## Ethics statement

Written informed consent was obtained from a parent or guardian on behalf of each child and the study was approved by the ethics committee of Beijing Children’s Hospital, Capital Medical University. Written informed consent to participate in this study was provided by the participants’ legal guardian/next of kin.

## Author contributions

All authors helped to perform the research; MJL wrote the manuscript; BC contributed to the project management; QL participated in the interpretation of data. QW, ML, XL, DW, WL, CS, and JC took part in the collection of clinical samples; CG conceived and designed the project as well as revised the manuscript. All listed authors revised the paper critically and approved the final version of the submitted manuscript.

## Funding

This study was funded by The Pediatric Medical Coordinated Development Center of Beijing Hospitals Authority (XTYB201808), the National Key Research and Development Program of China (2016YFC0901505, 2016YFC1305304), and the Beijing Municiple Administration of Hospital Clinical Medicine Development of Special Funding Support (No. ZYLX201821).

## Conflict of interest

The authors declare that the research was conducted in the absence of any commercial or financial relationships that could be construed as a potential conflict of interest.

## Publisher’s note

All claims expressed in this article are solely those of the authors and do not necessarily represent those of their affiliated organizations, or those of the publisher, the editors and the reviewers. Any product that may be evaluated in this article, or claim that may be made by its manufacturer, is not guaranteed or endorsed by the publisher.
